# ALDH1A1 in Cancers: Bidirectional Function, Drug Resistance, and Regulatory Mechanism

**DOI:** 10.3389/fonc.2022.918778

**Published:** 2022-06-22

**Authors:** Hanxun Yue, Zenan Hu, Rui Hu, Zeying Guo, Ya Zheng, Yuping Wang, Yongning Zhou

**Affiliations:** ^1^The First School of Clinical Medicine, Lanzhou University, Lanzhou, China; ^2^Department of Gastroenterology, The First Hospital of Lanzhou University, Lanzhou, China; ^3^Key Laboratory for Gastrointestinal Diseases of Gansu Province, The First Hospital of Lanzhou University, Lanzhou, China; ^4^Key Laboratory for Reproductive Medicine and Embryo of Gansu Province, The First Hospital of Lanzhou University, Lanzhou, China; ^5^College of Chemistry and Chemical Engineering, Lanzhou University, Lanzhou, China

**Keywords:** aldehyde dehydrogenases, cancer stem cell, carcinogenesis, drug resistance, hepatocellular carcinoma

## Abstract

Aldehyde dehydrogenases 1 family member A1(ALDH1A1) gene codes a cytoplasmic enzyme and shows vital physiological and pathophysiological functions in many areas. ALDH1A1 plays important roles in various diseases, especially in cancers. We reviewed and summarized representative correlative studies and found that ALDH1A1 could induce cancers *via* the maintenance of cancer stem cell properties, modification of metabolism, promotion of DNA repair. ALDH1A1 expression is regulated by several epigenetic processes. ALDH1A1 also acted as a tumor suppressor in certain cancers. The detoxification of ALDH1A1 often causes chemotherapy failure. Currently, ALDH1A1-targeted therapy is widely used in cancer treatment, but the mechanism by which ALDH1A1 regulates cancer development is not fully understood. This review will provide insight into the status of ALDH1A1 research and new viewpoint for cancer therapy.

## Introduction

Aldehyde dehydrogenases (ALHDs) are a group of cytoplasmic enzymes that use nicotinamide adenine dinucleotide (NAD) as a coenzyme to oxidize aldehydes into the corresponding carboxylic acids (1, 2). The human genome is thought to contain 19 functional ALDH genes, including ALDH1 family genes. ALDH1A1 is one of the main members of the ALDH1 family. The ALDH1A1 gene is located in subregion 13 of region 21, on the long arm of human chromosome 9. This gene encodes homotetrameric cytoplasmic proteins in various tissues. ALDH1A1 has a greater affinity for the oxidation of both all-trans and 9-cis-retinal molecules than ALDH1A2 and ALDH1A3 (3). ALDH1A has an NAD+ binding pocket (amino acids 8–135 and 159–270), a catalytic site (amino acids 271–470), and an oligomerization domain (amino acids 140–158 and 486–459). ALDH1A1 can exist in monomeric, dimeric, or tetrameric forms. The tetrameric and monomeric forms of ALDH1A1 are the most abundant. ALDH1A1 exists predominantly as a tetramer at high concentrations but the activity of its monomeric form is the highest (4). According to the GenBank database (https://www.ncbi.nlm.nih.gov/genbank/), ALDH1A1 was highly expressed in the liver, duodenum, and other tissues.

In the past decade, researchers have found that ALDH1A1 had vital physiological and pathophysiological functions in many systems, such as the central nervous system, as well as inflammatory and metabolic disorders (5–7). ALDH1A1 overexpression has been found to play an important role in obesity, diabetes, and other diseases (8–13). Because the retinoic acid (RA) signaling pathway is involved in the regulation of gene expression in cancer stem cells (CSCs), researchers have focused on the role of ALDH1A1 in cancers worldwide (14–16). Although several reviews on ALDH1A1 are available, no review has been published that discusses the roles of ALDH1A1 in all cancers (6, 17–19) (**Table 1**).

**Table 1:** Representative Studies of ALDH1A1 in Cancers.

Author	Year	Origin of organ	Relative molecular	Functional role	Ref.
Adam	2012	Brain	–	Tumor-suppressor	(20)
Okudela	2013	Lung	–	Tumor-suppressor	(21)
Wang	2013	Cervix	miR-23b	Oncogenic	(22)
Wilson	2013	Liver	–	Bidirectional	(23)
Patlolla	2013	Lung	–	Oncogenic	(24)
Xu	2014	Colorectum	β-catenin	Oncogenic	(25)
Cao	2014	Lung	–	Oncogenic	(26)
Xing	2014	Thyroid	–	Oncogenic	(27)
Li	2014	Stomach	MMP9	Oncogenic	(28)
Duong	2014	Pancreas	–	Oncogenic	(29)
Pandrangi	2014	Breast	–	Oncogenic	(30)
Liu	2015	Breast	–	Tumor-suppressor	(31)
Sjoüstroüm	2015	Breast	–	Bidirectional	(32)
Gao	2015	Lung	LGR5	Oncogenic	(33)
Hoshino	2015	Pancreas	SMAD4 TGF-β	Oncogenic	(34)
Condello	2015	Ovary	β-catenin EZH2 DDB2	Oncogenic	(35)
Tanaka	2015	Liver	AFP	Tumor-suppressor	(36)
Kesharwani	2015	Breast	–	Oncogenic	(37)
Yassin	2016	Lung	–	Oncogenic	(38)
Erfani	2016	Skin	–	Oncogenic	(39)
Wang	2016	Colorectum	–	Tumor-suppressor	(40)
Kim	2016	Colorectum	–	Bidirectional	(41)
Ma	2016	Lung	NOTCH3	Oncogenic	(42)
Yokoyama	2016	Breast	BRD4	Oncogenic	(43)
Januchowski	2016	Ovary	P-gp BCRP	Ocncogenic	(44)
Croker	2017	Breast	–	Oncogenic	(45)
Kalantari	2017	Prostate	–	Oncogenic	(46)
Sun	2017	Esophagus	CPA4	Oncogenic	(47)
Lu	2017	Esophagus	DDK1 SOX2	Oncogenic	(48)
Yu	2017	Lung	TAZ	Oncogenic	(49)
Wang	2017	Pancreas	AURKA	Oncogenic	(4)
Wang	2017	nasopharynx	β-catenin TCF4 NOR1 AKT GSK-β	Oncogenic	(50)
Duong	2017	Pancreas	NRF2	Oncogenic	(51)
Allison	2017	Breast	CYP2J2	Oncogenic	(52)
Yang	2017	Liver	–	Bidirectional	(53)
van der Waals	2018	Colorectum	–	Oncogenic	(54)
Yang	2018	Colorectum	–	Oncogenic	(55)
Xia	2018	Breast	–	Oncogenic	(56)
Tulake	2018	Cervix	OCT4	Oncogenic	(57)
Ye	2018	Stomach	–	Oncogenic	(58)
Wu	2018	Tonsillar	–	Tumor-suppressor	(59)
Ciccone	2018	Breast	VEGF HIF-1α	Oncogenic	(60)
Cui	2018	Ovary	DDB2 C/EBPβ	Oncogenic	(61)
Zhao	2018	Bladder	YAP	Oncogenic	(62)
Oria	2018	Pancreas	–	Oncogenic	(63)
Wang	2018	Breast	ERα36	Oncogenic	(64)
Kalra	2018	Breast	CYP2C19	Oncogenic	(65)
Roy	2018	Ovary	–	Oncogenic	(66)
Wang	2018	Breast	CXCR4 EpCAM MUC1	Oncogenic	(67)
Kwiatkowska	2018	Skin	–	Oncogenic	(68)
Liu	2019	Stomach	–	Oncogenic	(69)
Wanandi	2019	Breast	–	Oncogenic	(70)
Gong	2019	Stomach	miR-625	Oncogenic	(71)
Świerczewska	2019	Ovary	PTPRK	Oncogenic	(72)
Wang	2019	Lung	–	Oncogenic	(73)
Charkoftaki	2019	Colorectum	–	Oncogenic	(74)
Nwani	2019	Ovary	–	Oncogenic	(75)
Althobiti	2020	Breast	CD44 CD24 TWIST SOX9 EPCAM CD133	Oncogenic	(76)
Nagare	2020	Ovary	CD9 CD24 EPHA1	Oncogenic	(77)
Szafarowski	2020	Head & neck	–	Oncogenic	(78)
Yoshino	2020	Liver	ARID1A	Oncogenic	(79)
Namekawa	2020	Bladder	TUBB3	Oncogeinc	(80)
Jiang	2020	Prostate	RARα Est1	Oncogenic	(81)
Elcheva	2020	Blood	OXB4 MYB	Oncogenic	(82)
Wang	2020	Esophagus	AKT	Oncogenic	(83)
Tieng	2020	Colorectum	–	Oncogene	(84)
Kaipio	2020	Ovary	EGFR PI3K mTOR AURKA	Oncogenic	(85)
Liu	2020	Breast	BRD4	Oncogenic	(86)
Gyan	2021	Breast	CD44 CD24	Oncogenic	(87)
Wang	2021	Esophagus	β-catenin AKT1 Slug c-Myc Vimentin	Oncogenic	(88)
Liu	2021	Breast	TKA1 GM-CSF	Oncogenic	(89)
Narendra	2021	Blood	ZEB2 EZH2 MUC1 miR-16-5p miR-26a-5p	Oncogenic	(90)
Narendra	2021	Blood	ADMET	Oncogenic	(91)
Yamashita	2022	Lung	CD133 p53	Oncogenic	(92)
Nachiyappan	2022	Lung	EHMT1 C/EBPβ	Oncogenic	(93)
Zhou	2022	Bladder	YAP	Oncogenic	(94)
Okamoto	2022	Breast	–	Oncogenic	(95)

To understand the roles of ALDH1A1 in cancers, we reviewed and summarized representative correlative studies in this article. We summarized the consensus and controversies regarding the functions, regulatory mechanism, diagnostic value, and selective inhibitors of ALDH1A1. Based on the results of our experiments and bioinformatic assay, the potential uses of ALDH1A1 in hepatocellular carcinoma (HCC) will be discussed.

## ALDH1A1 Overexpression Is an Oncogenic Factor in Most Cancers

Since 2010, numerous studies have verified the fact that ALDH1A1 could promote tumor initiation and tumor progression. Several years ago, Yassin et al. had found that ALDH1A1 overexpression could reflect the poor historical subtype and advanced tumor grade in lung cancer patients (38). In a comparative study performed by Cao et al., the ALDH1A1 levels were found to be much higher in non-small cell lung cancer (NSCLC) patients at advanced stages than those with early-stage tumors (26). In contrast, triple−negative cases without ALDH1A1, CD133, and mutant p53 expression in lung adenocarcinomas were shown to have a much better prognosis than other cases (92).

The same is true for cancers of the digestive system. Li et al. found that ALDH1A1 overexpression was significantly associated with larger tumor size, deeper invasion, extensive lymph node metastasis, and advanced stage of gastric cancer. ALDH1A1 could represent an independent prognostic factor for both overall survival (OS) and recurrence-free survival (RFS) (28). ALDH1A1 overexpression was also a poor prognostic indicator of survival in patients with gastric neuroendocrine carcinoma (58). Liu et al. reported that ALDH1A1 overexpression was significantly associated with poorly differentiated histology in gastric cancer (69). Xu et al. found that ALDH1A1 overexpression had the same effect in patients with colorectal cancer as observed in gastric cancer (25, 54, 55). An analysis of the Oncomine database showed that ALDH1A1 was significantly upregulated in HCC tissues, compared to non-tumorous liver tissues (53). Peng et al. demonstrated that rs7852860 variants of the ALDH1A1 gene were associated with susceptibility to anti-tuberculosis drug-induced liver injury (96).

Extensive and in-depth studies on ALDH1A1 have been performed in breast cancer patients. First, Croker et al. determined the RNA expression of ALDH1A1 in breast cancer cells and found that ALDH1A1 overexpression contributed functionally the proliferation, adhesion, migration, extravasation, and micrometastasis of breast cancer (45). Althobiti et al. found that ALDH1A1 overexpression was associated with poor prognostic features, including an increased tumor grade, poor Nottingham prognostic index, extensive lymph node metastasis, and a greater extent of luminal B and triple-negative subtypes of breast cancer (76). In the African population, Gyan et al. found that ALDH1A1 was expressed at a high level in 90% of breast cancer specimens. This study further confirmed the increased oncogenicity of the CD44^+^/CD24^-^/ALDH1A1^+^ combination phenotype and its association with the increased tumor grade and clinical prognostic stage (87). Xia et al. conducted a population-based study to analyze the relationship between ALDH1A1 polymorphisms, alcohol consumption, and mortality among women diagnosed with breast cancer. They found that after adjusting all the results for multiple comparisons, rs7027604 was significantly associated with all-cause mortality in the rs1424482 CC genotype, and the rs7027604 AA genotype was positively associated with non-breast cancer mortality. Among long-term light drinkers, rs1888202 was associated with decreased all-cause mortality, while the association was not significant among non-drinkers or moderate/heavy drinkers. The increased risk of all-cause mortality associated with rs63319 was limited to women with a low level of native American ancestry (56). Furthermore, Wanandi et al. found that the expression of ALDH1A1 was higher in breast cancer stem cells than in the MCF-7 cell line, but was almost similar to that observed in the more aggressive cell line MDA-MB-231. These results suggested that ALDH1A1 overexpression might be related to the stemness and aggressiveness of breast cancers cells (70).

ALDH1A1 has been reported to promote cancers of the reproductive system. Nagare et al. found that most ALDH1A1-positive high-grade ovarian cancer cells resided in the G1 phase of the cell cycle. They also reported that the ALDH1A1-positive cells co-expressing the combination of CD9, CD24, or EPHA1 were more oncogenic and aggressive than ALDH1A1-negative cells (77). Tulake et al. found that ALDH1A1 and OCT4 were upregulated in both cervical squamous cell carcinoma and cervical intraepithelial neoplasia, as compared to healthy subjects. They found that ALDH1A1 expression levels were also increased in the peripherical blood obtained from cervical cancer patients; thus, ALDH1A1 expression could be regarded as an indicator of cervical cancer (57).

ALDH1A1 is also involved in the development of cancers that originate in other systems. A study involving Iranian prostate cancer patients by Kalantari et al. showed that the level of ALDH1A1 expression was positively correlated with tumor invasiveness (46). Among the most common cancer stem cell markers, only ALDH1A1 overexpression significantly affected the five-year OS of primary head and neck squamous cell carcinoma patients (78). ALDH1A1 levels were also higher in papillary thyroid carcinoma tissues than in normal thyroid tissues. ALDH1A1 overexpression was significantly associated with extrathyroidal extension and reflected a poorer RFS and distant recurrence-free survival (27). ALDH1A1 overexpression was also found to occur in skin cancers, particularly in melanomas (39).

## ALDH1A1 Acts as a Tumor Suppressor in Some Cancers

Although ALDH1A1 is seen as an oncogenic factor, it also exhibits different characteristics in some cancers. In a study performed by Adam et al., ALDH1A1 was co-expressed with GFAP and S100 in mature astrocytes and was a better prognostic marker for glioblastoma patients (20). In an experiment conducted by Wang et al., both cytoplasmic and nuclear expression levels were assessed in epithelial cells. Surprisingly, in the tissue microarray and whole-tissue cohorts, univariate analysis indicated that the cytoplasmic expression of ALDH1A1 cannot be considered a prognostic marker for colorectal cancers. Furthermore, nuclear expression levels of ALDH1A1 were significantly associated with longer disease-specific survival and nuclear expression levels in low-grade adenomas, and were predominantly higher than those in high-grade adenomas, primary colorectal cancer, and the corresponding liver metastases (40). Although ALDH1A1 is ubiquitously expressed in the liver, its function in HCC is still ambiguous. Tanaka et al. found that there was no significant difference in the ALDH1A1 level between HCC and non-cancerous liver tissues. In their study, the group with high ALDH1A1 levels was significantly associated with low serum levels of AFP, a small tumor diameter, low levels of lymphovascular invasion, a more differentiated pathology, and a less advanced stage (36). Yang et al. studied the relationship between ALDH1A1 and HCC using the GEO database and found high ALDH1A1 mRNA expression levels were significantly associated with longer 57–month recurrence-free survivals (53). Okudela et al. also found that the level of ALDH1A1 expression was negatively related to carcinogenesis in NSCLC patients. ALDH1A1 was remarkably downregulated in adenocarcinomas and large cell cancers. Among adenocarcinomas, the downregulation of ALDH1A1 tended to be more significant in high-grade, poorly differentiated tumors, and tumors with a stronger proliferating activity. Moreover, the incidence of this reduction was higher in smokers than in non-smokers (21). Liu et al. suggested that the high level of expression of ALDH1A1 mRNA in tumor tissues may be an independent predictor of favorable triple-negative breast cancer, based on an analysis performed using three databases and meta-analyses (31). Wu et al. reported that ALDH1A1-positive cells were a unique component of the crypt cellular microenvironment and were not stem cells. They also found that NGFR-positive and ALDH1A1-positive cells were lost during tumorigenesis with the expression of LGR5 in the tonsillar crypt niche; this may mark the breakdown of the normal microenvironment (59).

However, several groups of researchers believe that the behavior of ALDH1A1 is complex. In a study performed by Kim et al., ALDH1A1 overexpression decreased the proliferation and invasiveness of colorectal cancer cells, while colorectal cancer liver metastasis was more likely to occur in SW480/ALDH1A1-transfected mice (41). Sjoüstroüm et al. hypothesized that in breast cancer cells, ALDH1A1 overexpression was associated with either a better or a worse prognosis, depending on the cut-off. If weakly stained cells were considered to be positively stained, ALDH1A1 overexpression was associated with a better prognosis in two cohorts. If strongly stained cells were considered to be positively stained, ALDH1A1 overexpression was associated with a worse prognosis in one of the cohorts. In addition, stromal ALDH1A1 staining was associated with improved distant disease-free survival, and gene expression analysis showed that there was a relationship between ALDH1A1 overexpression and a favorable prognosis (32).

## Therapeutic Failure in Some Cancers Is Attributable to ALDH1A1-Induced Drug Resistance

Chemotherapy plays an important role in cancer treatment. However, many factors, including ALDH1A1, can cause chemotherapy failure. A study by Ma et al. showed that cisplatin induced NOTCH3 expression, and NOTCH3 overexpression was a prognostic factor for shorter progression-free survival and OS in NSCLC patients. They suggested that the chemoresistance of NSCLC patients was attributable to the promotion of ALDH1A1 expression by NOTCH3 and stimulation of autophagy (42). Wang et al. performed a UPLC−MS-based metabolomics analysis and revealed the metabolic dysregulation in lung adenocarcinoma. They found that the metabolic features of lung cancer cells were altered by ALDH1A1 overexpression, and levels of most metabolites, such as glucose-6-phosphate, fructose 1,6-diphosphate, propionyl-CoA, malic acid, phosphatidylcholine, glycerol phosphatidylcholine, GMP, citrulline, and arginine succinic acid were increased. These metabolites were involved in metabolic pathways such as glycolysis, the tricarboxylic acid cycle, glycerophospholipid metabolism, nucleotide metabolism, and the urea cycle. Among these, ALDH1A1 may amplify drug resistance in tumors through nucleotide metabolic pathways (73).

ALDH1A1-induced drug resistance is also common in cancers of the digestive system. Wang et al. found that ALDH1A1 promoted the development of resistance to 5-fluorouracil in esophageal squamous cell carcinoma (83). Oria et al. found that ALDH1A1 reduced the sensitivity of pancreatic cells toward gemcitabine and chemoradiation treatment (63). Fortunately, Duong et al. suggested that a therapeutic strategy involving a combination of dasatinib and gemcitabine might overcome gemcitabine resistance, as it would decrease the level of ALDH1A1 expression in pancreatic cancer. They also found that NRF2 could promote ALDH1A1 expression and the silencing of NRF2 could enhance the anti-proliferative effects of the chemotherapeutic agent 5-fluorouracil in pancreatic cancer cells (29, 51).

ALDH1A1 is highly expressed in breast cancer cells, and the knockdown of ALDH1A1 can significantly sensitize breast cancer cells to chemotherapy and radiotherapy (45). Wang et al. found that tamoxifen could promote ERα36 binding and the activation of estrogen-responsive elements in the ALDH1A1 promoter, to increase the transcription of ALDH1A1, which accounted for the resistance to hormone therapy and metastasis of breast cancer (64). In another study of breast cancer patients on adjuvant therapy, Kalra et al. found that CYP2C19 and ALDH1A1*2 (17 bp deletion), were significantly associated with the disease outcome, including OS, recurrence, and metastasis. Both these genes were involved in the pharmacokinetics of cyclophosphamide. Allison et al. hypothesized that ALDH1A1 was activated in the MDA-MB-468 breast cancer cell line, which stably expressed CYP2J2 and attenuated caspase-3/7 activity and the production of reactive oxygen species induced by cytotoxic agents, such as paclitaxel, doxorubicin, sorafenib, and staurosporin (52).

ALDH1A1-induced drug resistance also seriously hampered ovarian cancer and leukemia treatment. Data from a study performed by Nwani et al. demonstrated that ALDH1A1 was upregulated in ovarian cancer cells that survived exposure to platinum (75). Roy et al. found that ALDH1A1 overexpression was associated with a poor response to platinum-based therapy in patients with high-grade serous ovarian cancer (66). *via* Kaipio et al. found that ALDH1A1 expression was improved after neoadjuvant chemotherapy in high-grade serous ovarian cancers and that in treatment-naive tumors, ALDH1A1 overexpression was correlated with drug resistance and a reduced duration of survival. Notably, they mentioned that EGFR, PI3K-mTOR, and the AURKA inhibitor were toxic to cancer cells in tests that assessed drug sensitivity and resistance (85). Individuals with chronic myelogenous leukemia acquired resistance to cyclophosphamide, owing to the inactivation of its active metabolite aldophosphamide *via* the overexpression of ALDH1A1 (91).

## The Regulatory Mechanism of ALDH1A1 in Cancers Is Complex

**Figure 1:**
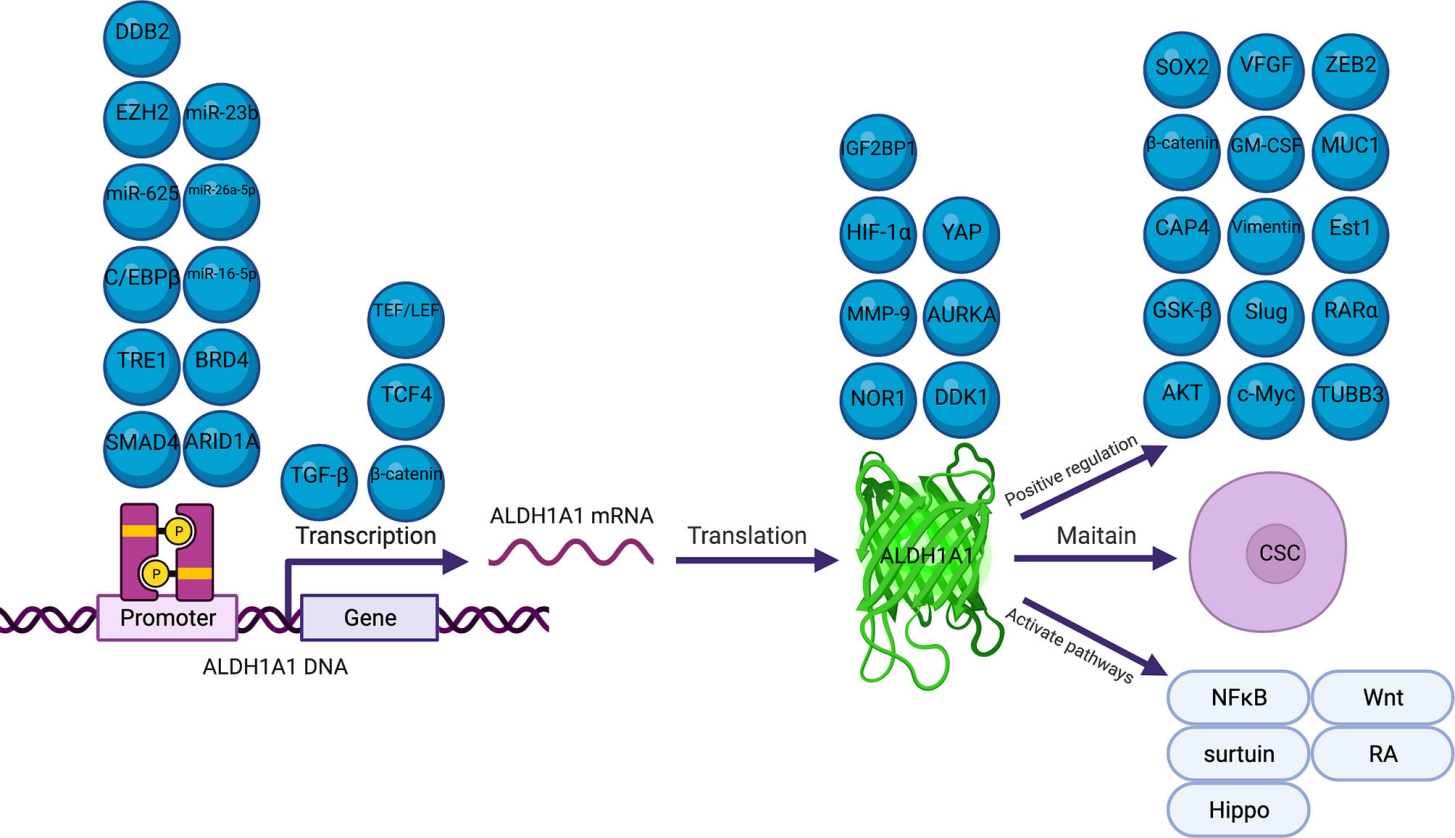
Molecules Involved in the Regulation of ALDH1A1.

Several previous studies have reported that many molecules and signal pathways are involved in the mechanism underlying ALDH1A1 regulation in cancers (28, 33, 47, 60, 72) (**Figure 1**). Our review described comprehensively the mechanisms of ALDH1A1 in the different processes of cancers and ALDH1A1 expression regulation. ALDH1A1 could induce cancers *via* the maintenance of CSC properties, modification of metabolism and promotion of DNA repair (43, 80, 81, 86). ALDH1A1 expression is regulated by several epigenetic processes, including, phosphorylation methylation, acetylation, methylation and miRNA modification (34, 71, 82, 90, 91).

CSCs have self-renewal capacity and differentiation potential and contribute to multiple tumor malignancies, such as recurrence, metastasis, heterogeneity, multidrug resistance, and radiation resistance (97). ALDH1A1 is thought to maintain CSC properties in a variety of cancers (49, 88). Yu et al. suggested that ALDH1A1 was a critical gene involved in the mediation of TAZ-induced lung tumorigenesis and CSC phenotypes. They found that TRE1, which was localized in the -256 ~ +52 region of the ALDH1A1 promoter, was majorly responsible for the activation of TAZ (49). Lu et al. suggested that DKK1 maintained the cancer stem-like properties of esophageal cancer cells *via* the ALDH1A1/SOX2 axis (48). Another group of researchers found that ALDH1A1 could also maintain esophageal CSC properties through promoting the stability of β-catenin and activating the AKT signal pathway (83). Wang et al. reported that the ectopic overexpression of NOR1 suppressed ALDH1A1 and β-catenin expression; β-catenin/TCF4 targeted the regulation of ALDH1A1 transcription; and the silencing of ALDH1A1 reduced AKT and GSK-β expression levels and resulted in the feedback inhibition of β-catenin expression. As a result, NOR1 could suppress the tumorigenic properties of CSCs in nasopharyngeal carcinoma *via* this signal circuit (50). The most recent study by Nachiyappan et al. showed that EHMT1 could promote tumor progression and maintain the stemness of alveolar rhabdomyosarcoma *via* the stabilization of C/EBPβ, which could activate the ALDH1A1 promotor (93).

As a metabolic enzyme, the effect of ALDH1A1 on metabolism plays an important role in cancer progression. Charkoftaki et al. tried to identify the role of ALDH1A1 in lipid metabolism in colon cancer cells using an integrated multi-omics approach. They found that the suppression of ALDH1A1 could downregulate oxidative phosphorylation, mitochondrial function, the sirtuin signaling pathway, and the retinol metabolism pathway. This approach provided greater insights into the pathways through which ALDH1A1 drives the development of cancers (74). Liu et al. made a breakthrough in research regarding the mechanism by which ALDH1A1 initiates breast cancer. They found that ALDH1A1 decreased the intracellular pH in breast cancer cells, in order to promote the phosphorylation of TAK1, activate the NFκB signal pathway, and increase the secretion of GM-CSF, and this led to myeloid-derived suppressor cell expansion and immunosuppression (89).

Phosphorylation is one of the most extensively and diligently studied posttranslational modifications which orchestrates a variety of cellular functions like cell growth, differentiation and apoptosis (98). Wang et al. showed that the phosphorylation-dependent regulation of ALDH1A1 was mediated by AURKA. They found AURKA could phosphorylate ALDH1A1 at the locations T267, T422, T439, at which phosphorylation primarily regulated ALDH1A1 activity. AURKA-mediated phosphorylation could rapidly facilitate the dissociation of tetrameric ALDH1A1 into a highly active monomeric species. Surprisingly, ALDH1A1 also reciprocates and prevents the degradation of AURKA, thereby triggering a positive activation loop that drives highly aggressive phenotypes in pancreatic cancer (4).

Acetylation influence a myriad of cellular and physiological processes, including transcription, phase separation, autophagy, mitosis, differentiation and neural function (99). In a study by Yoshino et al., chromatin immunoprecipitation assay showed that the level of H3K27 acetylation was significantly increased in the promoter region of ALDH1A1, while the HDAC1 level was significantly decreased in the ARID1A knocked-out cholangiocarcinoma cell line. Therefore, ARID1A may function as a tumor suppressor in cholangiocarcinoma through the transcriptional downregulation of ALDH1A1, along with a decrease in the levels of histone H3K27 acetylation (79).

The trimethylation of H3K27 was significantly correlated with the expression of ALDH1A1. In a study regarding ovarian cancer, Condello et al. identified that β-catenin could regulate ALDH1A1 *via* the TEF/LEF transcriptional complex, which was a key element of the Wnt signaling pathway. This mechanism could enhance spheroid formation in ovarian cancer cells. They reported that DDB2 recruited EZH2 to the ALDH1A1 promoter region, thereby facilitating the trimethylation of the local histone H3 at K27, and repressed the transcription of ALDH1A1 (35). Cui et al. also found that DDB2 could bind to the ALDH1A1 gene promoter and facilitate the enrichment of histone H3K27me3, and compete with the transcription factor C/EBPβ for binding to this region, eventually inhibiting the promoter activity of ALDH1A1. This mechanism involved the repression of ovarian cancer cell dedifferentiation (61).

MicroRNAs are small noncoding RNAs that regulate gene expression *via* recognition of cognate sequences and interference of transcriptional, translational or epigenetic processes (100). Bioinformatics analysis identified miR-16-5p and miR-26a-5p to be hub miRNAs for ALDH1A1 (91). The regulatory mechanism of ALDH1A1 was discussed in a study by Wang et al., who found that miR-23b could directly bind to the 3´-UTR region of ALDH1A1, to cause its reduction (22).

## ALDH1A1 Can be Seen as a Therapeutic Target For Cancers

Because ALDH1A1 is an oncogenic factor in many cancers, treatments targeting ALDH1A1 have become a research hotspot. During the diagnosis of NSCLC, the combined application of ALDH1A1 and carcinoembryonic antigen can significantly increase the sensitivity, compared to that observed with the use of carcinoembryonic antigen alone (26). Okamoto et al. developed a new long-acting fluorescence probe that could identify breast cancer stem cells *via* the targeting of ALDH1A1 (95).

Many researchers have developed specific ALDH1A1-targeting drugs that could be used to treat cancer (**Table 2**). Some of them believe that the combination of anti-ALDH1A1 therapy and chemotherapy can offset the ALDH1A1-induced drug resistance in cancer patients. Patlolla et al. showed that β-escin could inhibit tobacco carcinogen-induced lung tumor formation by modulating ALDH1A1-positive cells (24). Silybin could efficiently inhibit the proliferation, invasion, and metastasis of prostate cancer cells, by reducing ALDH1A1 expression levels (81). In 2015, Kesharwani et al. reported a new approach for overcoming drug resistance to breast chemotherapy *via* the targeting of synthetic curcumin analogs against ALDH1A (37). Wang et al. found that quercetin could inhibit the proliferation, clonal expansion, and mammsophere formation of CD44+/CD24- breast cancer stem cells by inhibiting ALDH1A1, CXCR4, EpCAM, and MUC1 (67). Pandrangi et al. found that ellipticine, a plant alkaloid, could inhibit mammosphere formation in ALDH1A1 overexpressed breast cancer stem cells, whereas paclitaxel enhanced mammosphere formation in the same cell lines (30). Condello et al. discovered a novel ALDH1A1 small molecule inhibitor named A37, which could moderately sensitize ovarian cancer cells to cisplatin (35). Liu et al. reported that NCT-501, an ALDH1A1 selective inhibitor, could augment the efficacy of olaparib during ovarian cancer treatment (86). Another group of researchers reported that the ALDH1A1 inhibitor disulfiram and chemotherapeutic agent gemcitabine cooperatively inhibited breast tumor growth and tumorigenesis by purging ALDH+ tumor-initiating cells and activating T-cell immunity (89). Recently, benzimidazole derivatives have been found to act as potent and selective ALDH1A1 inhibitors (8). It has been proven that N42 could also selectively bind to and inhibit ALDH1A1 (9).

**Table 2:** Proven Inhibitors of ALDH1A1.

Name	Structure	MW	IC_50_ (μg)	Cancer cell type	Ref.
A37/CM37	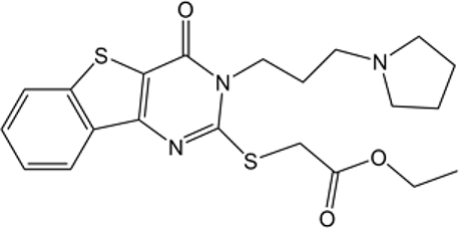	431.6	4.6 ± 0.8	Ovarian cancer	(75, 101)
BDC	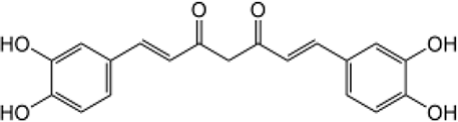	No accurate data	No accurate data	Breast cancer	(37)
Benzimidazole derivatives-61	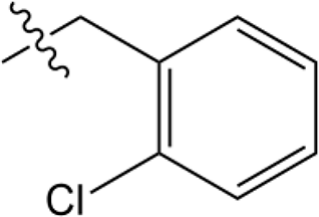	78.34 ± 7.42	10.23 ± 0.28	-	(8)
Benzimidazole derivatives-65	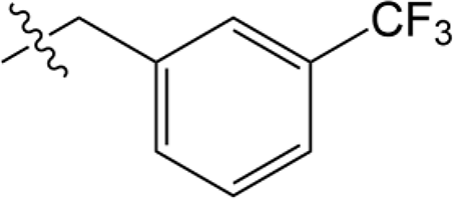	82.07 ± 7.9	0.921 ± 0.19	-	(8)
Disulfiram	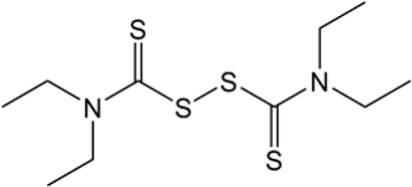	296.54	No accurate data	Breast cancer	(89)
Ellipticine	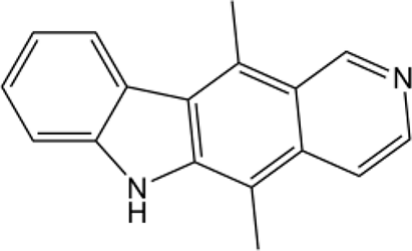	246.31	No accurate data	Breast cancer	(30)
NCT-501	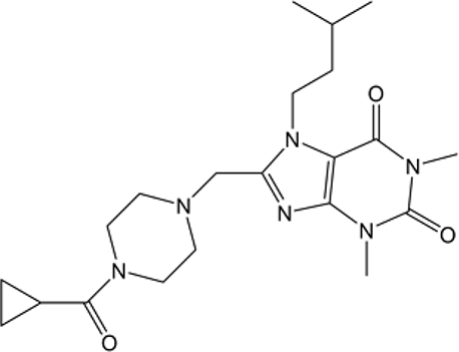	416.52	40	Ovarian cancer	(86)
Quercetin	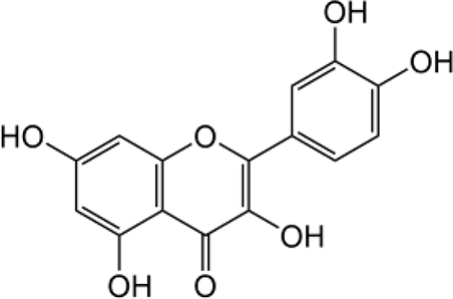	302.24	No accurate data	Breast cancer	(67)
Silybin	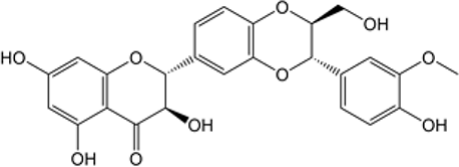	482.44	68	Prostate cancer	(81)
β-Escin	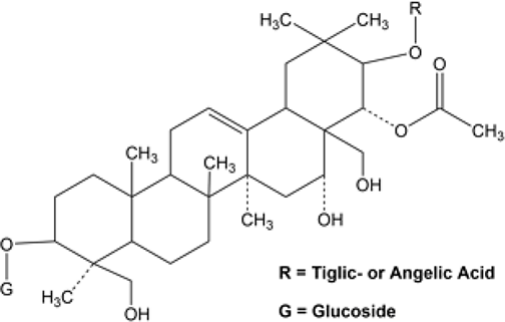	1131.26	No accurate data	Non-small lung cancer	(102)

Several ALDH1A1-based cancer prevention and treatment measures have been developed. The oligomer-dependent activity of ALDH1A1 signifies that the targeting of its oligomerization state may be an efficient therapeutic approach that could counteract its protective functions in cancer (4). Januchowski et al. found that ATRA treatment could lead to the downregulation of ALDH1A1, P-gp, and BCRP proteins (44). Yokoyama et al. showed that JQ1, one of the bromodomain and extra-terminal inhibitors, suppressed BRD4-mediated ALDH1A1 expression through a super-enhancer element and its associated enhancer RNA. They also found that the combination of JQ1 and cisplatin could improve the survival of mice with ovarian cancer (43). Moreover, an allogeneic, whole-cell, genetically modified therapeutic melanoma vaccine could generate immune responses to ALDH1A1 and improve long-term survival in advanced melanoma patients (68).

## Conclusions and Future Perspectives

In this review, we summarize and discuss studies that examined the roles of ALDH1A1 in cancers. Over the past decade, researchers have discovered that ALDH1A1 could induce cancers *via* the maintenance of cancer stem cell properties, modification of metabolism, promotion of DNA repair. ALDH1A1 expression is regulated by several epigenetic processes. ALDH1A1 also acted as a tumor suppressor in certain cancers. ALDH1A1 is highly expressed in the liver, and this is attributable to its bidirectional functions in HCC. The detoxification of ALDH1A1 often causes chemotherapy failure. Currently, ALDH1A1-targeted therapy is widely used in cancer treatment, but the mechanism by which ALDH1A1 regulates cancer development is not fully understood. In our previous studies, we observed that ALDH1A1 expression was significantly improved in HCC and the knockdown of ALDH1A1 weakened the proliferation and invasion of the Huh-7 cell line. Unlike in other cancers, ALDH1A1 did not maintain the properties of liver CSCs (23). Therefore, we believe that ALDH1A1 can induce HCC by mechanisms other than those involving CSCs. In clinical practice, the detoxification of ALDH1A1 may prolong the survival of HCC patients. This hypothesis needs to be tested in future studies. In the future, we will continue to explore the roles of ALDH1A1 in cancers, especially in HCC.

## Author Contributions

HY and ZH accomplished the manuscript and equally contributed to this study. YNZ and YW designed this study. YZ, RH and ZG provided guidance for this study. All authors contributed to the article and approved the submitted version.

## Funding

This work was supported by the Foundation of the First Hospital of Lanzhou University, China (ldyyyn2021-3), the Open Fund of Gansu Provincial Key Laboratory for Biotherapy and Regenerative Medicine (zdsyskfkt-201705).

## Conflict of Interest

The authors declare that the research was conducted in the absence of any commercial or financial relationships that could be construed as a potential conflict of interest.

## Publisher’s Note

All claims expressed in this article are solely those of the authors and do not necessarily represent those of their affiliated organizations, or those of the publisher, the editors and the reviewers. Any product that may be evaluated in this article, or claim that may be made by its manufacturer, is not guaranteed or endorsed by the publisher.
